# Editorialets

**Published:** 1908-10

**Authors:** 


### Editorialets.

THE FIRST NORTHER.

Now fades the glimmering straw hat out of sight,
And all the clothes a wint'ry aspect wear,—
Save where ye editor,—always in a tight,—
Must wear his linen duster still,—or go bare.

Please remit!

---

DR. AND MRS. H. C. GHENT (Belton, Texas), announce the marriage of their daughter, Sarah, to Mr. Chamblin C. Carter, October 6th, inst.

---

CONFOUND THE PRINTER.—We wrote "the great doctor expelled a hat-full of gallstones from a woman," and the confounded printer got it "a pot-full." Provoking, isn't it?

---

"IS THE DOCTOR IN?" inquired a caller at Dr. Bibbs' office, of the young "cullud-lady" (new) attendant.

"N-a-w, sir; he's done gone out to the infirmity."

---

NEW RESIDENCE FOR QUARANTINE OFFICER AT GALVESTON.—The old quarantine station at Galveston is to be replaced by an $8000 building for the office and residence of the inspector.

---

THE (CONTINUED) REPORT of the Corpus Christi meeting of health officers does not appear this month because Dr. Bibb, the secretary, failed to furnish copy. He has gone to the Tuberculosis Congress.

---

DR. J. HICKS FLORENCE, State Quarantine Inspector at Galveston, is in charge of the State Public Health Department at Austin during the absence of State Health Officer Brumby at Washington in attendance upon the Tuberculosis Congress.

---

CORRECTION.—The North Texas District Medical Society will meet in Dallas, December 8th and 9th, on which occasion the great surgeons, Mayo and Ferguson, will be the guests, instead of the Southern Surgical Association, as incorrectly stated in our last issue.

---

DR. AND MRS. FRANK PASCHAL (San Antonio, Texas), announce the marriage of their daughter, Bettie, to Mr. George Robert Sanders, October 14th, inst.

We acknowledge the courtesy of an invitation to both the above weddings, and extend congratulations to the fortunate gentlemen.

---

THE inimitable and only Lydston, the "Live Wire of Chicago," who writes such red-hot, snappy, sparkling and spontaneous epistles to the marines in the "Red Back," articles that attract so much attention, has been invited to be the guest of the Texas State Medical Association at Galveston. He will deliver an address, Lydstonian in character.

---

I WANT TO SELL good house, cost $3000; contract practice with two coal mines that pay forty to sixty dollars per month, two miles from town; population of town, 2800; good schools, churches; six mines in operation that work seven hundred to eight hundred men. Three doctors, good opening. Will sell for $2500. Wish to go west on account of wife's health. Address Box 118, Rockdale, Texas.

---

DR. GEORGE DOCK, of the University of Michigan, formerly of the Medical Department of the University of Texas, has accepted the chair of medicine in Tulane University at New Orleans. Dr. Dock is well known to the Texas profession, with whom he is a great favorite, and, in the opinion of the "Red Back," he is the Osler of the future. He will attend our next State Association meeting at Galveston, May, 1909, and will be our guest.

---

THE MEDICAL PRACTICE ACT SUSTAINED:

"Waco, Sept. 18.—A decision of State-wide importance was handed down today by Judge Surratt in the district court refusing Dr. S. A. Morse a license as a physician. Morse sought a mandamus to compel the State Board of Medical Examiners to grant him a license. The Board had refused, alleging unprofessional conduct. Morse claimed the statute providing for such action was invalid, but Judge Surratt upholds the law. The plaintiff says he will appeal."—*Statesman.*

---

H. V. C.—The success which attends the conjunctive employment of Viburnum Opulus, Dioscorea Villosa and Scutellaria Lateriflora as presented in Hayden's Viburnum Compound for the treatment of diseases of women, is due as much to the quality of each individual drug as it is to their proper proportioning; hence, it is seldom, if ever, possible to secure ideal results by the extemporaneous combining of such specimens as are procurable in the open market.

If it has once satisfactorily served you in your practice, it will do so again, provided you prescribe the original H. V. C. and see that a substitute is not administered.

---

MR. FAIRCHILD HONORED.—"For the first time in several years in this city the degree of Master of Pharmacy was conferred upon five distinguished men from different sections of the United States, who have attained distinction in the art of preparing medicines and drugs. Those who received this honor were Samuel W. Fairchild, of New York; Horatio Nelson Fraser, of New York; John F. Hancock, of Baltimore; S. A. D. Sheppard, of Boston, and William McIntyre, of Philadelphia."—*Evening Bulletin,* Philadelphia.

Samuel W. Fairchild is treasurer of the old and famous firm of Fairchild Bros. & Foster, of New York, whose preparations of pepsin and panopeptine are standard.—EDITOR.

---

"PROSERPINE" AGAIN: A WARM NUMBER.—In our next issue there will be another communication from "Proserpine" (Queen of Hades), alias "Mrs. Pluto," on the same subject as that which in our May number created such a sensation. It is a review, or criticism, of Professor Malchow's book, "Sexual Life." In a letter accompanying this communication the writer authorizes us to give her real name. She is a well-known society woman of Houston, active in humanitarian work, a college graduate, a lady of cultivation and refinement,—a mother and a grandmother. She challenges the doctors to controvert her position anent the marital relations of man and woman.

---

Transverse scalp wounds require comparatively many sutures, longitudinal wounds but few.—*American Journal of Surgery.*

